# Systemic Inflammatory Response Syndrome in Patients Hospitalized for Acute Decompensation of Cirrhosis

**DOI:** 10.1155/2021/5581587

**Published:** 2021-04-25

**Authors:** Ariane Borgonovo, Caroline Baldin, Dariana C. Maggi, Livia Victor, Emilia T. O. Bansho, Juliana Piedade, Letícia M. Wildner, Lívia Guimarães, Maria L. Bazzo, Tamires Rocha, Esther B. Dantas-Corrêa, Camila Alcântara, Flávia Fernandes, Janaina L. Narciso-Schiavon, Gustavo H. S. Pereira, Leonardo L. Schiavon

**Affiliations:** ^1^Division of Gastroenterology, Department of Internal Medicine, Federal University of Santa Catarina, Florianópolis, Santa Catarina, Brazil; ^2^Department of Gastroenterology and Hepatology, Bonsucesso Federal Hospital, Rio de Janeiro, Brazil; ^3^Department of Clinical Analysis, Federal University of Santa Catarina Clinical Analysis Laboratory, University Hospital Polydoro Ernani de São Thiago, Federal University of Santa Catarina, Florianópolis, Santa Catarina, Brazil

## Abstract

**Background:**

Although recently challenged, systemic inflammatory response syndrome (SIRS) criteria are still commonly used in daily practice to define sepsis. However, several factors in liver cirrhosis may negatively impact its prognostic ability. *Goals*. To investigate the factors associated with the presence of SIRS, the characteristics of SIRS related to infection, and its prognostic value among patients hospitalized for acute decompensation of cirrhosis. *Study*. In this cohort study from two tertiary hospitals, 543 patients were followed up, up to 90 days. Data collection, including the prognostic models, was within 48 hours of admission.

**Results:**

SIRS was present in 42.7% of the sample and was independently associated with upper gastrointestinal bleeding (UGB), ACLF, infection, and negatively related to beta-blockers. SIRS was associated with mortality in univariate analysis, but not in multiple Cox regression analysis. The Kaplan–Meier survival probability of patients without SIRS was 73.0% and for those with SIRS was 64.7%. The presence of SIRS was not significantly associated with mortality when considering patients with or without infection, separately. Infection in SIRS patients was independently associated with Child-Pugh C and inversely related to UGB. Among subjects with SIRS, mortality was independently related to the presence of infection, ACLF, and Child-Pugh C.

**Conclusions:**

SIRS was common in hospitalized patients with cirrhosis and was of no prognostic value, even in the presence of infection.

## 1. Introduction

Cirrhosis is the final stage of liver diseases from different etiologies, characterized by nodular regeneration and liver fibrosis [1]. Forty percent of patients with cirrhosis may be asymptomatic and may remain that way for more than a decade, but progressive deterioration is usually observed when complications such as ascites, variceal hemorrhage, encephalopathy, hepatorenal syndrome, and infections arise. In the decompensated stage of cirrhosis, 5-year mortality is around 50%, with 70% of these deaths directly attributable to liver disease [2].

Liver cirrhosis is characterized by several systemic abnormalities, including cirrhosis-associated immune dysfunction, a condition associated with both systemic inflammation and immunosuppression [3]. As a result, infections are among the most relevant clinical problems in patients with cirrhosis. Bacterial infections are present at admission in about one-third of the patients [4, 5] and are related to significant morbidity, mortality, and progression with acute-on-chronic liver failure (ACLF) [4, 6, 7]. Consequently, early identification of patients with cirrhosis at high risk of complications and mortality related to infections is decisive for an effective management.

For many years, systemic inflammatory response syndrome (SIRS) was used to define sepsis. Nevertheless, SIRS criterion was recognized to be limited as a prognostic tool in general population [8] and, particularly, among patients with cirrhosis [5, 9]. Several factors commonly observed among cirrhotics may impair SIRS parameters, including tachypnea due to encephalopathy, hypersplenism-related leukopenia, or bradycardia induced by beta-blockers. Recently, the Sepsis-3 criterion was proposed as new definitions of sepsis in general population and subsequently validated in patients with cirrhosis [5, 10, 11]. One of the most important limitations of these new criteria is that, in the context of a patient with cirrhosis recently admitted and without a baseline SOFA score available, Sepsis-3 criterion is of little value [5]. In addition, even outside the context of cirrhosis, these new definitions are not unanimously accepted [12]. For that reason, SIRS criterion is still commonly used in daily practice and its clinical significance in patients with cirrhosis is not completely known. Therefore, the aim of this study was to investigate the factors associated with the presence of SIRS, the characteristics of SIRS related to infection, and its prognostic value among patients recently hospitalized for acute decompensation of cirrhosis.

## 2. Materials and Methods

### 2.1. Patients

This was a prospective cohort study that evaluated patients admitted in two Brazilian tertiary hospitals (University Hospital of Polydoro Ernani São Thiago of Florianópolis, SC and Federal Hospital Bonsucesso, Rio de Janeiro, RJ) due to acute decompensation cirrhosis (AD), between January 2011 and October 2015. Subjects in the following situations were excluded: (1) hospitalization for elective procedures; (2) hospitalization for less than 48 hours; (3) admissions not related to complications of liver cirrhosis; (4) hepatocellular carcinoma outside Milan criteria; (5) extrahepatic malignancy; (6) severe extrahepatic disease; (7) use of immunosuppressive drugs; and (8) human immunodeficiency viruses (HIV) infection.

The diagnosis of cirrhosis was established either histologically (when available) or by the combination of clinical, imaging, and laboratory findings in patients with evidence of portal hypertension.

The study protocol complies with the ethical principles of the Declaration of Helsinki and was approved by the Ethics Committee on Human Research from the two institutions involved in the study. Informed consent was obtained from all participants or their surrogates.

## 3. Methods

AD was defined as the acute development of hepatic encephalopathy, large ascites, gastrointestinal bleeding, bacterial infection, or any combination of these [4].

Patients were evaluated in the first 48 hours of hospitalization by one of the researchers involved in the study, and the following clinical variables were collected: age, gender, etiology of cirrhosis, previous and current complications of cirrhosis, use of beta-blockers, and mean arterial pressure (MAP). Patients were followed during their hospital stay and thirty and 90-day mortality was evaluated by phone call, in case of hospital discharge.

All subjects admitted for acute decompensation of cirrhosis in the hospitals involved in the study are actively screened for bacterial infections. Diagnostic paracentesis was performed in all patients with ascites. Spontaneous bacterial peritonitis (SBP) was diagnosed when the neutrophil count of the ascitic fluid was ≥250 neutrophils/mm^3^ in the absence of intra-abdominal source of infection, regardless of negative culture [13]. Criteria for diagnosing other infections than SBP were adapted from the Centers for Disease Control and Prevention [14]. Hepatic encephalopathy was graded according to the West-Haven criteria [15] and if it was present, a precipitant event was actively investigated and lactulose was initiated and the dose adjusted as needed. All subjects with acute variceal bleeding received intravenous vasoactive drugs (terlipressin or octreotide) and an antibiotic (either oral norfloxacin or intravenous ceftriaxone) and underwent urgent therapeutic endoscopy after stabilization [16]. The severity of liver disease was estimated by the Child-Pugh classification system [17] and MELD (Model for End-Stage Liver Disease) [18].

SIRS was defined by the presence of at least two among the following criteria: body temperature <36°C or >38°C, heart rate >90 beats per minute (bpm), respiratory rate >20/min, white blood cells (WBC) < 4.000/*μ*L or >12.000/*μ*L, or immature neutrophils >10% [19]. ACLF was defined as proposed by the European Association for the Study of the Liver-Chronic Liver Failure (EASL-CLIF) Consortium [4].

### 3.1. Statistical Analysis

The normality of the variable distribution was determined by the Kolmogorov–Smirnov test. Continuous variables were compared using Student's *t*-test in the case of normal distribution or Mann–Whitney test in the remaining cases. Categorical variables were evaluated by the chi-square test or Fisher's exact test as appropriate. Multiple logistic regression analysis (enter method) was used to investigate the factors independently associated with the presence of SIRS and with infection among patients with SIRS. Univariate and multivariate Cox regression analyses (enter method) were used to investigate the association between the variables and survival. The Kaplan–Meier curves were used to illustrate survival according to two strata. All tests were performed by the MedCalc software, version 19.1 (MedCalc Software, Mariakerke, Belgium). A *P* value of less than 0.05 was considered statistically significant.

## 4. Results

### 4.1. Characteristics of the Sample

**Figure 1:**
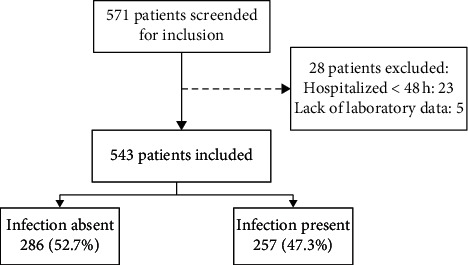
Flow-chart of the patients evaluated for inclusion, reasons for exclusion, and the final sample according to the presence of infection at initial evaluation.

Between January 2011 and October 2015, 571 patients were screened for inclusion. Twenty-three patients were excluded because they were hospitalized for less than 48 hours and five due to lack of laboratory data (Figure 1). Therefore, the final sample was composed of 543 patients, 287 from the state of Santa Catarina and 256 from the state of Rio de Janeiro.  Table 1 exhibits the characteristics of the included patients. The mean age was 55.4 ± 12.7 years, 64.5% were male, and the most common etiologic factor of cirrhosis was alcohol abuse (47.1%) followed by hepatitis C (40.1%). Upon admission, upper gastrointestinal bleeding was observed in 28.5% of cases, ascites in 60.2%, and hepatic encephalopathy in 45%. Bacterial infections were present in 47.3% of the sample. The most common bacterial infection was spontaneous bacterial peritonitis (10.5%) followed by skin infections (9.4%), urinary tract infection (9.2%), and pneumonia (8.7%). Infections without identified focus and less common types of infection, including bacterascites, and primary bacteraemia, accounted for 8.1% and 3.5% of the cases, respectively.

### 4.2. Factors Associated with the Presence of SIRS

Table 1 exhibits the comparison between patients with and without SIRS. SIRS was present in 232 patients (42.7%) and was associated with alcoholic etiology of cirrhosis (52.2% vs. 43.4%, *P* = 0.043), upper gastrointestinal bleeding (UGB) (33.6% vs. 24.8%; *P* = 0.024), hepatic encephalopathy (52.2% vs. 39.7%, *P* = 0.004), bacterial infection (62.5% vs. 36.0%, *P* < 0.001), and a lower proportion of individuals taking beta-blockers (50.2% vs. 37.3%, *P* = 0.003). Patients with SIRS also presented lower mean sodium (134.0 ± 6.3 vs. 135.1 ± 5.4 mEq/L, *P* = 0.032) and albumin (2.3 ± 0, 6 vs. 2.5 ± 0.6 mg/dL, *P* = 0.029), and higher median INR (1.5 vs. 1.4, *P* = 0.029), total bilirubin (2.5 vs. 1.9 mg/dL, *P* = 0.038), creatinine (1.2 vs. 1.0 mg/dL, *P* = 0.004), and CRP (39.1 vs. 6.3 mg/dL, *P* < 0.001). Patients with SIRS also had a higher proportion of Child-Pugh C (50.9% vs. 37.0%, *P* = 0.001) and a higher MELD score (18.5 ± 7.9 vs. 16.3 ± 6.0, *P* < 0.001).

A logistic regression analysis investigating factors independently associated with SIRS was performed including the following variables with *P* < 0.05 in the bivariate analysis: beta-blockers use, alcoholic etiology, UGB, infection, sodium, Child-Pugh C, and ACLF. Other variables with statistical significance in the bivariate analysis, such as hepatic encephalopathy, creatinine, albumin, total bilirubin, INR, and MELD, were not included in the regression analysis because they are already included or closely related to the Child-Pugh score and ACLF definition. In this analysis, SIRS was associated with UGB (OR 2.811, 95% CI 1.765–4.478; *P* < 0.001), ACLF (OR 1.688, 95% CI 1.064–2.676; *P* = 0.026), beta-blockers (OR 0.598, 95% CI 0.405–0.881; *P* = 0.009), and infection (OR 3.721, 95% CI 2.433–5.698; *P* < 0.001).

### 4.3. Prognostic Value of SIRS among Patients Hospitalized for Acute Decompensation of Cirrhosis

**Figure 2:**
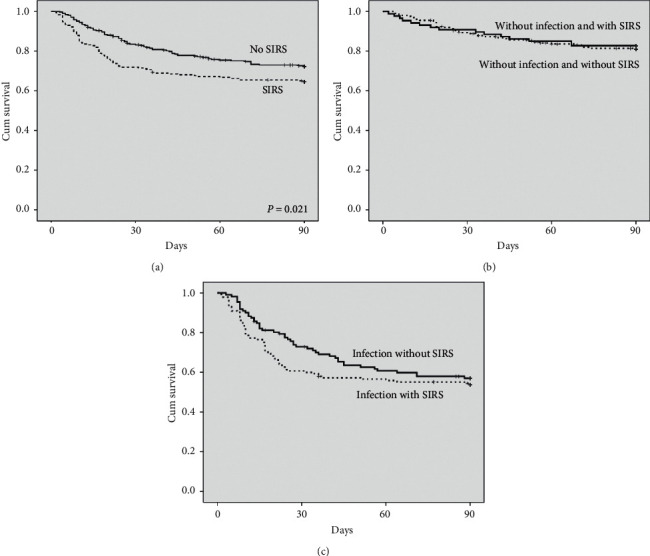
Cumulative 90-day survival of patients with cirrhosis according to the presence of SIRS. When considering the entire cohort, the 90-day survival probability was 73.0% for patients with SIRS and 64.7% for those without it (a). Among patients without infection, the 90-day Kaplan–Meier survival probability was 82.8% in subjects without SIRS and 81.4% in those with SIRS (b). SIRS criterion was also applied in patients with infection and the survival probability was 58.0% in subjects not fulfilling SIRS criterion and 53.8% among those who fulfill it (c).

Among all the individuals included in the study, 108 (19.9%) died within 30 days and 166 (30.6%) died within 90 days of hospitalization.  Table 2 shows the comparison between survivors and nonsurvivors. Ninety-day mortality was associated with ascites, hepatic encephalopathy, infection, ACLF, Child-Pugh C, SIRS, and inversely related to UGB. Mortality was also related to lower sodium and albumin levels, and higher INR, total bilirubin, creatinine, leukocyte count, CRP, and MELD. The following variables were included in a multivariate Cox regression analysis: UGB, infection, Child-Pugh C, ACLF, and SIRS criteria. In this analysis, infection (HR = 1.968, IC 95% 1.371–2.826, *P* < 0.001), Child-Pugh C (HR = 2.401, IC 95% 1.671–3.448, *P* < 0.001), and ACLF (HR = 2.824, IC 95% 2.024–3.939, *P* < 0.001) were independently related to 90-day survival. SIRS was not associated with mortality in the multivariate analysis (HR = 1.016, IC 95% 0.736–1.403, *P* = 0.923). The Kaplan–Meier survival probability of patients without SIRS was 73.0% and for those with SIRS was 64.7% (Figure 2(a)) (*P* = 0.021). Survival was evaluated according to the presence or absence of SIRS in patients infected or not. In this analysis, the Kaplan–Meier survival probability of patients without infection was similar, irrespectively of the presence of SIRS (81.4% vs. 82.8%, *P* = 0.742) (Figure 2(b)). Interestingly, even among patients with infection, similar 90-day survival was observed for patients with and without SIRS (58.0% vs. 53.8%, *P* = 0.313) (Figure 2(c)).

### 4.4. Factors Associated with the Presence of Infection among Patients with SIRS

In this analysis including only patients with SIRS, when compared to patients without infection, infected subjects exhibited a higher proportion of patients with ascites (73.1% vs. 43.7%, *P* < 0.001), hepatic encephalopathy (57.2% vs. 43.7%, *P* = 0.045), ACLF (42.0% vs. 20.7%, *P* = 0.001), Child-Pugh C (61.3% vs. 33.3%, *P* < 0.001), and a lower proportion of UGB (19.3% vs. 57.5% *P* < 0.001). Patients with infection also had higher mean MELD (20.2 ± 8.2 vs. 15.6 ± 6.4, *P* < 0.001), higher median INR (1.5 vs. 1.4, *P* < 0.001), total bilirubin (3.0 mg/dL vs. 1.6 mg/dL, *P* = 0.001), creatinine (1.3 mg/dL vs. 1.0 mg/dL, *P* = 0.003), CRP (14.1 mg/L vs. 5.6 mg/L, *P* = 0.005), and lower mean serum sodium (132.9 ± 6.5 vs. 135.9 ± 29.2 mEq/L, *P* = 0.007). No relationship was observed between infection and other studied variables, including beta-blockers use (Table 3).

A logistic regression analysis was performed including only SIRS patients and with infection as a dependent variable. This analysis included the following covariates: UGB, serum sodium, ACLF, and Child-Pugh C. Again, variables already included or closely related to the Child-Pugh score and ACLF definition were not included in the regression analysis. CRP was not included in this analysis given the high number of missing values (57 cases). Infection among patients with SIRS was independently associated with Child-Pugh C (OR 2.227, 95% CI 1.147–4.325; *P* = 0.018) and inversely related to UGB (OR 0.210, 95% CI 0.111–0.397; *P* < 0.001).

### 4.5. Factors Associated with Prognosis among Cirrhotic Patients with SIRS

**Figure 3:**
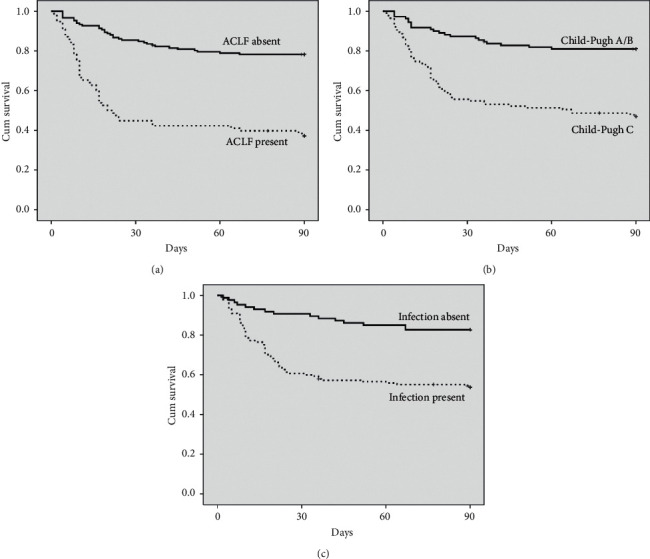
Factors associated with 90-day survival among patients with SIRS. The 90-day survival probability was 78.3% for patients without ACLF and 37.2% for with ACLF (a). Survival was 81.1% among Child-Pugh A/B patients and 47.0% among Child-Pugh C (b). Infection was also strongly related to lower survival among SIRS patients (53.8% vs. 82.8%, *P* < 0.001) (c).

When only patients with SIRS were analyzed, univariate Cox regression showed that survival was related to the presence of ascites, HE, bacterial infection, sodium, albumin, INR, total bilirubin, creatinine, leukocyte count, CRP, MELD score, ACLF, and Child-Pugh C (Table 4). A multivariate Cox regression analysis was performed including the following variables: infection, ACLF, Child-Pugh C, and serum sodium. In this analysis, survival was independently related to infection (HR = 2.135, IC 95% 1.200–3.800, *P* = 0.010), ACLF (HR = 2.837, IC 95% 1.782–4.516, *P* < 0.001), and Child-Pugh C (HR = 2.243, IC 95% 1.324–3.803, *P* = 0.003). Among patients with SIRS, the Kaplan–Meier survival probability of patients without ACLF was 78.3% and for those with ACLF was 37.2% (Figure 3(a)) (*P* < 0.001). Similarly, 90-day survival was 81.1% among Child-Pugh A/B subjects and 47.0% among Child-Pugh C (Figure 3(b)) (*P* < 0.001). Infection was also strongly related to lower survival among SIRS patients (53.8% vs. 82.8%, *P* < 0.001) (Figure 3(c)).

## 5. Discussion

Cirrhosis is characterized by a persistent inflammatory state that can be highly exacerbated during acute insults, especially bacterial infections [20]. However, even in the absence of clinically apparent bacterial infections, cirrhosis complications are related to an increase in bacterial translocation, contributing to an increase in the proinflammatory phenotype, possibly with systemic consequences [20, 21]. Therefore, SIRS is a common event in patients with cirrhosis admitted for acute decompensation.

In the present study, SIRS was present in 42.7% of the patients and was independently associated with ACLF, infection, and UGB and inversely related to beta-blockers. The connection between SIRS, infection, and ACLF is expected, as bacterial infections are the most common precipitant factors of both SIRS and ACLF, and the two conditions are associated with systemic inflammation and organ dysfunction [4, 22, 23]. UGB can be associated with findings of SIRS by promoting clinical and laboratory abnormalities that can mimic systemic inflammation. However, infection is a common complication of patients with cirrhosis hospitalized for UGB and also a precipitant factor for variceal bleeding [24, 25]. Nevertheless, this is unlikely in our cohort of patients recently hospitalized in whom the frequency of bacterial infection was lower in those with UGB. In the present study, beta-blockers were inversely related to SIRS. It was previously shown that chronic beta-blockers' use is associated with improvement in intestinal permeability, reduced bacterial translocation, and lower risk of infections in cirrhotic patients [26, 27]. In addition, beta-blockers were also associated with decreased rates of sepsis [27] and improved survival of patients with acute-on-chronic liver failure [28]. However, no association between beta-blockers and survival was observed in the present study, suggesting that this supposed protective effect on SIRS development was not reflected in better prognosis. One possible explanation is that beta-blockers can lower heart rate, decreasing the proportion of patients that fulfill SIRS criteria, without exerting any other significant benefit in this context.

The presence of SIRS was associated with higher mortality in univariate Cox regression analysis. However, when evaluating according to the presence or absence of infection, no prognostic impact of SIRS was observed. In previous studies evaluating the prognostic significance of SIRS among patients with cirrhosis, SIRS was a frequent complication, ranging from 14% to 41% of the cases, and universally associated with worse prognosis [29–35]. However, in the vast majority of cases, SIRS was strongly related to infection and no comparison between patients with SIRS according to the presence of infection was performed. In two recent studies aimed at validating Sepsis-3 criteria and qSOFA in patients with cirrhosis, SIRS was associated with worse survival in univariate analysis, but not multivariate Cox regression [5, 36]. These data suggest that infection appears to be the real prognostic factor in patients hospitalized for acute decompensation of cirrhosis. SIRS criteria are of little value, if any, in determining prognosis and defining sepsis among these individuals.

As bacterial infections are commonly seen in patients with SIRS, an analysis was performed comparing patients with SIRS with and without infection. In this analysis, Child-Pugh C was independently related to the presence of infection, while admission for UGB was related to the absence of infection. There are no previous studies evaluating factors related to the presence of infection specifically among cirrhotic patients with SIRS. However, the severity of cirrhosis is associated with the risk and prognosis of bacterial infections, and also infections can further deteriorate liver function [37]. Regarding the inverse relationship between infections and UGB, these results could be partially explained by the routine use of prophylactic antibiotics that decreases significantly the infection rate [25]. However, the most obvious explanation is that infections are naturally more frequent, at least early during hospitalization, among other presentations of acute decompensation, such as hepatic encephalopathy and rapid worsening of ascites.

Among patients with SIRS, mortality was independently associated with infection, ACLF, and Child-Pugh C. Although the presence of SIRS was associated with some peculiar characteristics, prognostic factors among SIRS patients mirror those of subjects with cirrhosis without SIRS. Infections are more frequent among patients with SIRS and are importantly related to prognosis in cirrhosis [38, 39]. Similarly, ACLF is a frequent complication of advanced cirrhosis, commonly triggered by infection, and strongly related to mortality [40]. Therefore, as observed for cirrhotics in general, among patients hospitalized for acute decompensation of cirrhosis who developed SIRS, the prognosis is related to the severity of the acute insult and presence of organ failure.

In conclusion, SIRS is commonly observed among patients recently hospitalized for acute decompensation of cirrhosis, even in the absence of infections. SIRS without infection was frequently related to UGB and was of no prognostic value. Even in patients with infection, the presence of SIRS was not associated with higher mortality. These data indicate that SIRS criterion is of no value in determining prognosis or in defining sepsis among patients with cirrhosis and its use should be discouraged in clinical practice.

## Figures and Tables

**Table 1:** Characteristics of included patients and comparison according to the presence of SIRS.

	All (*n* = 543)	SIRS absent (*n* = 311)	SIRS present (*n* = 232)	*P*
Age (years), mean ± SD	55.4 ± 12.7	56.1 ± 12.3	54.4 ± 13.0	0.133
Male gender, (%)	64.5	64.0	65.1	0.791
Diabetes, (%)	40.9	44.1	37.4	0.196
Beta-blockers, (%)	44.7	50.2	37.3	0.003

Etiology of cirrhosis (%)				
Hepatitis C	40.1	38.6	42.2	0.390
Hepatitis B Alc	5.4	6.1	4.3	0.362
Alcohol	47.1	43.4	52.2	0.043
Autoimmune hepatitis	3.7	4.2	3.0	0.477
Others	21.4	20.9	22.0	0.761

Complication at admission (%)				
Ascites	60.2	58.8	62.1	0.257
Hepatic encephalopathy	45.0	39.7	52.2	0.004
Gastrointestinal bleeding	28.5	24.8	33.6	0.024
Bacterial infection (%)	47.3	36.0	62.5	<0.001

Laboratory data				
Sodium (mEq/L), mean ± SD	134.6 ± 5.8	135.1 ± 5.4	134.0 ± 6.28	0.032
Albumin (g/dL), mean ± SD	2.4 ± 0.6	2.5 ± 0.6	2.3 ± 0.6	0.029
INR, median	1.5	1.4	1.5	0.029
Total bilirubin (mg/dL), median	2.1	1.9	2.5	0.038
Creatinine (mg/dL), median	1.1	1.0	1.2	0.004
Leukocyte count (x10^9^), median	6.40	6.27	6.90	0.191
CRP (mg/L), median	7.3	6.3	39.1	<0.001
MELD score, mean ± SD	17.2 ± 7.0	16.3 ± 6.0	18.5 ± 7.9	<0.001
ACLF (%)	26.3	20.7	33.9	0.001

Child-Pugh classification				
A	7.0	7.3	6.6	0.757
B	50.0	55.7	42.5	0.003
C	43.0	37.0	50.9	0.001
MAP (mmHg), mean ± SD	86.4 ± 15.4	86.8 ± 15.7	86.0 ± 15.0	0.552

SIRS = systemic inflammatory response syndrome; SD = standard deviation; INR = international normalised ratio; CRP = C-reactive protein; MELD = Model for End-Stage Liver Disease; ACFL = acute-on-chronic liver failure; MAP = mean arterial pressure.

**Table 2:** Comparison of demographic, clinical, and laboratory data according to 90-day survival.

	Survivors (*n* = 377)	Nonsurvivors (*n* = 166)	HR (95% CI)	*P*
Age (years), mean ± SD	54.7 ± 12.8	57.0 ± 12.2	1.012 (0.999–1.025)	0.065
Male gender (%)	63.4	66.9	1.139 (0.825–1.574)	0.429
Diabetes (%)	39.9	43.2	1.124 (0.772–1.636)	0.543
Beta-blockers (%)	45.9	41.9	0.876 (0.640–1.199)	0.409

Complication at admission (%)				
Ascites	50.9	81.3	3.450 (2.334–5.100)	<0.001
Hepatic encephalopathy	33.9	22.9	2.021 (1.483–2.775)	<0.001
Gastrointestinal bleeding	38.8	59.0	0.577 (0.394–0.844)	0.005
SIRS (%)	39.8	49.4	1.428 (1.053–1.936)	0.022
Bacterial infection (%)	20.7	40.4	2.995 (2.156–4.159)	<0.001

Laboratory data				
Sodium (mEq/L), mean ± SD	135.6 ± 5.1	132.4 ± 6.6	0.927 (0.905–0.949)	<0.001
Albumin (g/dL), mean ± SD	2.5 ± 0.6	2.2 ± 0.6	0.455 (0.353–0.586)	<0.001
INR, median	1.4	1.7	1.880 (1.614–2.189)	<0.001
Total bilirubin (mg/dL), median	1.7	3.2	1.071 (1.054–1.089)	<0.001
Creatinine (mg/dL), median	1.0	1.5	1.343 (1.257–1.436)	<0.001
Leukocyte count (x10^9^), median	6.11	7.47	1.055 (1.033–1.077)	<0.001
CRP (mg/L), median	6.0	10.5	1.006 (1.004–1.008)	<0.001

MELD score, mean ± SD	14.9 ± 5.2	22.4 ± 7.5	1.119 (1.100–1.138)	<0.001
ACLF (%)	14.7	52.4	4.321 (3.181–5.870)	<0.001
Child-Pugh C (%)	31.1	69.3	3.817 (2.734–5.330)	<0.001
MAP (mmHg), mean ± SD	87.2 ± 14.9	84.6 ± 16.3	0.996 (0.984–1.008)	0.518

HR = hazard ratio; SD = standard deviation; SIRS = systemic inflammatory response syndrome; INR = international normalised ratio; CRP = C-reactive protein; MELD = Model for End-Stage Liver Disease; ACFL = acute-on-chronic liver failure; MAP = mean arterial pressure.

**Table 3:** Factors associated with the presence of infection among patients with SIRS.

	SIRS without infection (*n* = 87)	SIRS with infection (*n* = 145)	*P*
Age (years), mean ± SD	53.5 ± 11.9	55.0 ± 13.7	0.803
Male gender (%)	66.7	64.1	0.696
Diabetes (%)	35.1	39.4	0.563
Beta-blockers (%)	38.4	36.6	0.791

Complication at admission (%)			
Ascites	43.7	73.1	<0.001
Hepatic encephalopathy	43.7	57.2	0.045
Gastrointestinal bleeding	57.5	19.3	<0.001

Laboratory data			
Sodium (mEq/L), mean ± SD	135.9 ± 29.2	132.9 ± 6.5	0.007
Albumin (g/dL), mean ± SD	2.5 ± 0.6	2.2 ± 0.6	<0.001
INR, median	1.4	1.5	<0.001
Total bilirubin (mg/dL), median	1.6	3.0	0.001
Creatinine (mg/dL), median	1.0	1.3	0.003
Leukocyte count (x10^9^), median	6.07	8.10	0.008
CRP (mg/L), median	5.6	14.1	0.005
MELD score, mean ± SD	15.6 ± 6.4	20.2 ± 8.2	<0.001
ACLF (%)	20.7	42.0	0.001

Child-Pugh classification			<0.001
A	11.9	3.5	0.014
B	54.8	35.2	0.004
C	33.3	61.3	<0.001
MAP (mmHg), mean ± SD	87.3 ± 13.9	85.2 ± 15.7	0.304

SIRS = systemic inflammatory response syndrome; SD = standard deviation; INR = international normalised ratio; CRP = C-reactive protein; MELD = Model for End-Stage Liver Disease; ACFL = acute-on-chronic liver failure; MAP = mean arterial pressure.

**Table 4:** Comparison of demographic, clinical, and laboratory data according to 90-day survival among patients with SIRS (*n* = 232).

	Survivors (*n* = 150)	Nonsurvivors (*n* = 82)	HR (95% CI)	*P*
Age (years), mean ± SD	54.0 ± 13.9	55.2 ± 11.4	1.004 (0.988–1.021)	0.608
Male gender (%)	64.7	65.9	1.048 (0.664–1.654)	0.842
Diabetes (%)	36.9	38.3	1.061 (0.630–1.785)	0.825
Beta-blockers (%)	38.0	35.9	0.914 (0.576–1.452)	0.704

Complication at admission (%)				
Ascites	52.0	80.5	2.935 (1.699–5.073)	<0.001
Hepatic encephalopathy	36.7	28.0	1.576 (1.013–2.451)	0.043
Gastrointestinal bleeding	48.0	59.8	0.742 (0.458–1.202)	0.225
Bacterial infection, (%)	52.0	81.7	3.328 (1.900–5.831)	<0.001

Laboratory data				
Sodium (mEq/L), mean ± SD	135.1 ± 5.8	132.2 ± 6.5	0.945 (0.916–0.976)	0.001
Albumin (g/dL), mean ± SD	2.5 ± 0.7	2.1 ± 0.5	0.454 (0.322–0.639)	<0.001
INR, median	1.4	1.6	1.583 (1.294–1.937)	<0.001
Total bilirubin (mg/dL), median	2.0	3.6	1.069 (1.046–1.092)	<0.001
Creatinine (mg/dL), median	1.0	1.7	1.265 (1.161–1.379)	<0.001
Leukocyte count (x10^9^), median	6.07	9.03	1.049 (1.024–1.075)	<0.001
CRP (mg/L), median	8.5	14.9	1.005 (1.002–1.008)	0.002

MELD score, mean ± SD	15.8 ± 6.0	23.3 ± 8.4	1.094 (1.070–1.120)	<0.001
ACLF (%)	19.6	59.8	4.176 (2.679–6.512)	<0.001
Child-pugh C (%)	37.5	74.4	3.604 (2.192–5.924)	<0.001
MAP (mmHg), mean ± SD	85.7 ± 14.5	84.6 ± 16.8	0.995 (0.981–1.010)	0.526

SIRS = systemic inflammatory response syndrome; HR = hazard ratio; SD = standard deviation; INR = international normalised ratio; CRP = C-reactive protein; MELD = Model for End-Stage Liver Disease; ACFL = acute-on-chronic liver failure; MAP = mean arterial pressure.

## Data Availability

Data are available on request from the authors
